# GP‐2250, a novel anticancer agent, inhibits the energy metabolism, activates AMP‐Kinase and impairs the NF‐kB pathway in pancreatic cancer cells

**DOI:** 10.1111/jcmm.17825

**Published:** 2023-06-30

**Authors:** Britta Majchrzak‐Stiller, Marie Buchholz, Ilka Peters, Daniel Waschestjuk, Johanna Strotmann, Philipp Höhn, Stephan Hahn, Chris Braumann, Waldemar Uhl, Thomas Müller, Hanns Möhler

**Affiliations:** ^1^ Department of General and Visceral Surgery St. Josef‐Hospital, Ruhr‐University Bochum Bochum Germany; ^2^ Department of Molecular Gastrointestinal Oncology Ruhr‐University Bochum Bochum Germany; ^3^ Department of General, Visceral and Vascular Surgery, Evangelische Kliniken Gelsenkirchen Akademisches Lehrkrankenhaus der Universität Duisburg‐Essen Gelsenkirchen Germany; ^4^ Geistlich Pharma AG Lucerne Switzerland; ^5^ Institute of Pharmacology University of Zurich and Department of Chemistry and Applied Biosciences, Swiss Federal Institute of Technology (ETH) Zurich Switzerland

**Keywords:** aerobic glycolysis, apoptosis, GP‐2250, NFκB, pancreatic cancer, proliferation, ROS

## Abstract

GP‐2250, a novel anticancer agent, severely limits the energy metabolism, as demonstrated by the inhibition of hexokinase 2 and glyceraldehyde‐3‐phosphate dehydrogenase and a decrease of ATP. Rescue experiments with supplementary pyruvate or oxaloacetate demonstrated that a TCA cycle deficit largely contributed to cytotoxicity. Activation of the energy‐deficit sensor, AMP‐dependent protein kinase, was associated with increased phosphorylation of acetyl‐CoA carboxylase and Raptor, pointing to a possible deficit in the synthesis of fatty acids and proteins as essential cell components. Binding of p65 to DNA was dose‐dependently reduced in nuclear lysates. A transcriptional deficit of NF‐κB (nuclear factor kappa‐light‐chain‐enhancer of activated B cells) was substantiated by the downregulation of cyclin D1 and of the anti‐apoptotic Bcl2, in line with reduction in tumour cell proliferation and induction of apoptosis, respectively. The upregulation of p53 concomitant with an excess of ROS supported apoptosis. Thus, the anticancer activity of GP‐2250 is a result of disruption of energy metabolism and inhibition of tumour promotion by NF‐κB.

## 1 INTRODUCTION

**FIGURE 1:**
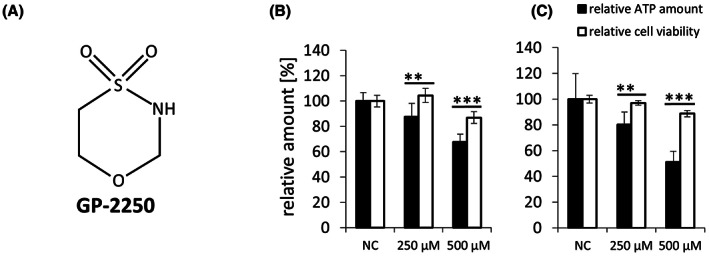
Impact of GP‐2250 on ATP level. Structure of GP‐2250 (A). Decrease of ATP in BxPC3 cells and (B) Panc Tul cells (C) compared to cell viability (MTT test) following incubation with GP‐2250 (250 and 500 μM) for 6 h. The level of ATP and cell viability were assessed in the same test samples. MTT, 3‐(4,5‐dimethylthiazol‐2‐yl)‐2,5 diphenyltetrazoliumbromid; NC, negative control.**p ≤ 0.01; ***p ≤ 0.001.

The oxathiazinane compound GP‐2250 (Figure 1A) is a highly effective antineoplastic agent, as has been demonstrated for pancreatic adenocarcinoma in vitro and in vivo.^1^, ^2^ GP‐2250 inhibited tumour cell proliferation and induced apoptotic cytotoxicity. In vivo, the growth of patient‐derived pancreatic tumour tissue was strongly inhibited in xenograft mouse models.^1^, ^2^ GP‐2250 is presently in a clinical trial for the treatment of pancreatic cancer (NCT 03854110).^3^ Its molecular mechanism of action, however, has remained unclear.

As GP‐2250 showed comparable antineoplastic activity in pancreatic and mesothelioma tumour cell lines,^1^, ^4^ GP‐2250 was expected to target a common feature of cancer cells such as aerobic glycolysis, a hallmark of cancer metabolism.^5^, ^6^, ^7^ As the first step in glycolysis, hexokinase (HK) was a potential target for GP‐2250, with HK2 being highly expressed in cancer cells. HK2 couples metabolic and proliferative effects by rewiring metabolism to aerobic glycolysis and protects against pro‐apoptotic stimuli by docking to mitochondria.^8^ Glyceraldehyde‐3‐phosphate dehydrogenase (GAPDH) is the rate‐limiting enzyme of aerobic glycolysis thereby exerting control over glycolytic energy metabolism.^9^ Tumour cells contain functional mitochondria^5^, ^7^ and inhibition of the trichloroacetic acid cycle (TCA cycle) can likewise contribute to an energy deficit either as a downstream consequence of the impaired glycolysis or by direct inhibition of TCA cycle enzymes such as α‐ketoglutarate dehydrogenase (αKGDH).

Inhibition of glycolytic enzymes and TCA cycle by GP‐2250 would be expected to reduce ATP and trigger an energy crisis. Activation of the energy‐deficit sensor, adenosine monophosphate‐dependent protein kinase (AMPK), suppresses tumour growth^10^ by introducing a metabolic austerity programme via inhibition of downstream ATP‐consuming biosynthetic pathways.^11^, ^12^ They include the synthesis of fatty acids, proteins and nucleotides, key building blocks for cell growth and proliferation. Tumour growth can also be limited by inhibition of the transcription factor NF‐κB (nuclear factor kappa‐light‐chain‐enhancer of activated B cells), a major driver of tumour progression with a key impact on cell cycle regulation and apoptosis. Its inhibition would be expected to downregulate the expression of cyclin D1, the driver of mitosis, and Bcl2, an anti‐apoptotic survival factor.^13^ The drug‐induced oxidative reactive oxygen species (ROS) stress can be expected to upregulate the transcription factor p53, which provides additional anti‐tumour activity. Thus, by targeting energy metabolism and the NF‐κB pathway of tumour cells, GP‐2250 would be expected to display a dual approach to cytotoxicity.

## 2 MATERIALS AND METHODS

### 2.1 Cell lines and culture conditions

Two human pancreatic cancer cell lines were used: BxPC3^14^ (ATCC—LGC Standards GmbH) and Panc TuI (ATCC—LGC Standards GmbH). Authentication was analysed by STR analysis. Panc TuI cells^15^ were cultured in Dulbecco's Modified Eagle Medium (DMEM 25 mM Glucose). BxPC3 were maintained in RPMI 1640. Both cultures were supplemented with the antibiotics penicillin (100 U/mL), streptomycin (100 U/mL) and 2 mM L‐glutamine. Cells were grown as monolayer and cultured in 25 cm^2^ flasks at 37°C and 5% CO_2_ in a humidified atmosphere.

### 2.2 Western blot analysis

Protein isolation for western blotting^16^ was carried out by RIPA (radioimmunoprecipitation assay) lysis (Abcam) and the BCA (bicinchoninic acid) assay (Thermo scientific). After loading equal amounts of protein per lane (30 μg protein), 7%–20% PROTEAN‐TGX (Tris‐Glycine eXtended) gels (BIO RAD), were electrophoresed at 250 V for 30–45 min and transferred on to a Trans‐Blot Turbo PVDF (polyvinylidenfluorid) membrane (BIO RAD) using a Trans‐Blot Turbo system (BIO RAD). Thereafter, the membranes were blocked in EveryBlot Blocking Buffer (BIO RAD) according to the manufacturer's antibody specification protocol for 5 min and incubated overnight at 4°C with the respective primary antibody [P‐AMPKalpha Rabbit mAB (T172) #2535, P‐ACC (Ser79) Rabbit mAB #11818, P‐Raptor (Ser792) Rabbit mAB #2083, p53 Rabbit mAB #2527, mTOR Rabbit Ab #2972, Akt Rabbit Ab #9272, cyclin D1 Rabbit mAB #55506, beta‐actin Rabbit mAB #8457, α‐tubulin Rabbit mAb #2125, Bcl2 Rabbit mAb #4223 and BAX Rabbit AB #2772 (CST)] at 1:1000 dilution. The membranes were washed using PBST (phosphate buffered saline + tween 0.025%) and incubated with an Anti‐rabbit IgG HRP‐linked AB 7074; (1:2000 CST). ChemiDoc MP Imaging System (BIO RAD) was used for band detection. Western blots were done in triplicate and representative blots are depicted.

### 2.3 ATP and cell viability determination

The ATP level in the cell lines was measured using the Luminescent ATP Detection Assay Kit (Abcam; ab1113849) following the manufacturer's instructions. The viability of cells was determined with the MTT assay as described earlier.^1^ The assay was performed in at least three independent experiments with consecutive passages.

### 2.4 HK2 assay

A HK activity assay kit (Merck, D, MAK091‐1KT)^17^ was used following the manufacturer's instructions. Cells were incubated with different concentrations of GP‐2250 for 24 h before lysis. To analyse HK2 activity, lysates were partly incubated for 1 h at 45°C to eliminate HK2, which is sensitive to temperature. The difference between total HK activity (lysates on ice) and HK 1 activity (lysates for 1 h at 45°C) reveals the activity of HK2.

### 2.5 rhHK2 enzyme activity assay

Recombinant human hexokinase 2 (rhHK2) enzyme activity was tested in vitro using HK2 from a HK2 inhibitor assay kit (Abcam, ab211114) following the manufacturer's instructions.

### 2.6 GAPDH enzyme activity assay

Following incubation with GP‐2250 for 24 h, cell lysates were prepared in lysis buffer [20 mM Tris (tris [hydroxymethyl] aminomethane) pH 7.8, 100 mM NaCL, 1% triton, protease inhibitors] followed by centrifugation. Enzyme activity was measured from 10 μL of cell lysate as described by Kornberg et al.^18^ Assays were performed in 10 mM sodium pyrophosphate buffer (pH 8.5) in 96‐well plates. Cell lysates were incubated with 20 mM sodium arsenate (made fresh on the day of experiment), 1 mM NAD+, and 2.88 mM glyceraldehyde‐3‐phosphate (G3P). Enzyme activity was measured using a microplate reader—spectrophotometer (Tecan) as the increase in absorbance at 340 nm is due to the reduction of NAD+. The assay was performed at 37°C. The lysate was first diluted into sodium pyrophosphate buffer to a volume of 100 μL. An additional 100 μL of reaction mix containing the sodium arsenate, NAD+ and G3P was added. Absorbance was measured every 5 min for 60 min.

### 2.7 rhGAPDH enzyme activity assay

Recombinant human GAPDH (rhGAPDH) (Abcam, ab 82633) was used. 2 μg of rhGAPDH was transferred to HEN buffer (250 mM Hepes‐NaOH, 1 mM EDTA 0 and 1 mM neocuproine) and incubated with PBS or GP‐2250 (100 and 250 μM) at 37°C. After 0, 30 and 60 min of incubation, an aliquot was taken for the enzyme assay. The assay was conducted using the GAPDH Activity Assay Kit (Abcam Cambridge, UK, ab204732) following the manufacturer's instructions.^19^

### 2.8 PDH enzyme activity assay

A pyruvate dehydrogenase (PDH) activity assay kit (Merck, D, MAK183‐1KT) was used following the manufacturer's instructions. Cells were incubated with different concentrations of GP‐2250 for 24 h before lysis.

### 2.9 αKGDH activity assay

An α‐ketoglutarate dehydrogenase (αKGDH) Activity Assay Kit (Merck, D, MAK189‐1KT) was used following the manufacturer's instructions.^20^ Cells were incubated with different concentrations of GP‐2250 for 24 h before lysis.

### 2.10 Rescue experiments with pyruvate and oxaloacetate

Cells were incubated with different concentrations of GP‐2250 for 24 h in culture medium supplemented or not supplemented with either pyruvate (PYR) (5 mM in DMEM with 2 mM Glucose) or oxaloacetate (OAA) (5 mM in DMEM with 25 mM Glucose). The viability of cells was determined with the MTT assay as described earlier.^1^ The assay was performed in at least three independent experiments with consecutive passages.

### 2.11 ROS assay

ROS was analysed using the Cellular ROS/Superoxide Detection Assay Kit (ab139476, Abcam)^21^ following the manufacturer's instructions.

### 2.12 NF‐κB transcription factor binding assay

The transcription factor NF‐κB was assayed with the NFκB p65 Transcription Factor Assay Kit (Abcam, ab133112)^22^ following the manufacturer's instructions. Lysates were prepared either from drug‐treated cells or, alternatively, from untreated cells with the lysates being directly incubated with GP‐2250. The nuclear extracts were prepared with the Nuclear Extraction Kit (Abcam, ab113474) according to the manufacturer's instructions.

### 2.13 Statistics and calculations

Results of MTT test, ATP assay, ROS assay, HK2 assay, GAPDH assay, PDH assay, NF‐κB assay, western blots and rescue experiments are expressed as mean ± SD. Comparison between experimental groups with normal distribution was performed using one‐way anova followed by Tukey's post hoc test and pairwise tests performed using *t*‐tests. *p* ≤ 0.05 were considered statistically significant and indicated in the figures as follows: *p ≤ 0.05; **p ≤ 0.01; ***p ≤ 0.001.

## 3 RESULTS

### 3.1 Induction of energy deficiency

The hypothesis that GP‐2250 induces an energy deficit was tested by measuring the level of ATP in the pancreatic tumour cell lines BxPC3 and Panc Tul. At the lowest dose tested (250 μM GP‐2250), a significant decrease in ATP was already apparent at 6 h in both BxPC3 and Panc Tul cells (12.4% ± 2.4% and 19.8% ± 2.3%, respectively), with no loss of cell viability (Figure 1B,C). A further decrease in ATP was apparent at the same time point with 500 μM GP‐2250 in both cell lines (32.5% ± 3.1% and 48.9% ± 4.8%, respectively), at which dose only a minor decrease of cell viability of 10%–12% was observed (Figure 1B,C). These findings of a drug‐induced deficit of ATP pointed to an inhibition of energy metabolism by GP‐2250.

### 3.2 Cytotoxicity by inhibition of glycolytic enzymes and the TCA cycle

**FIGURE 2:**
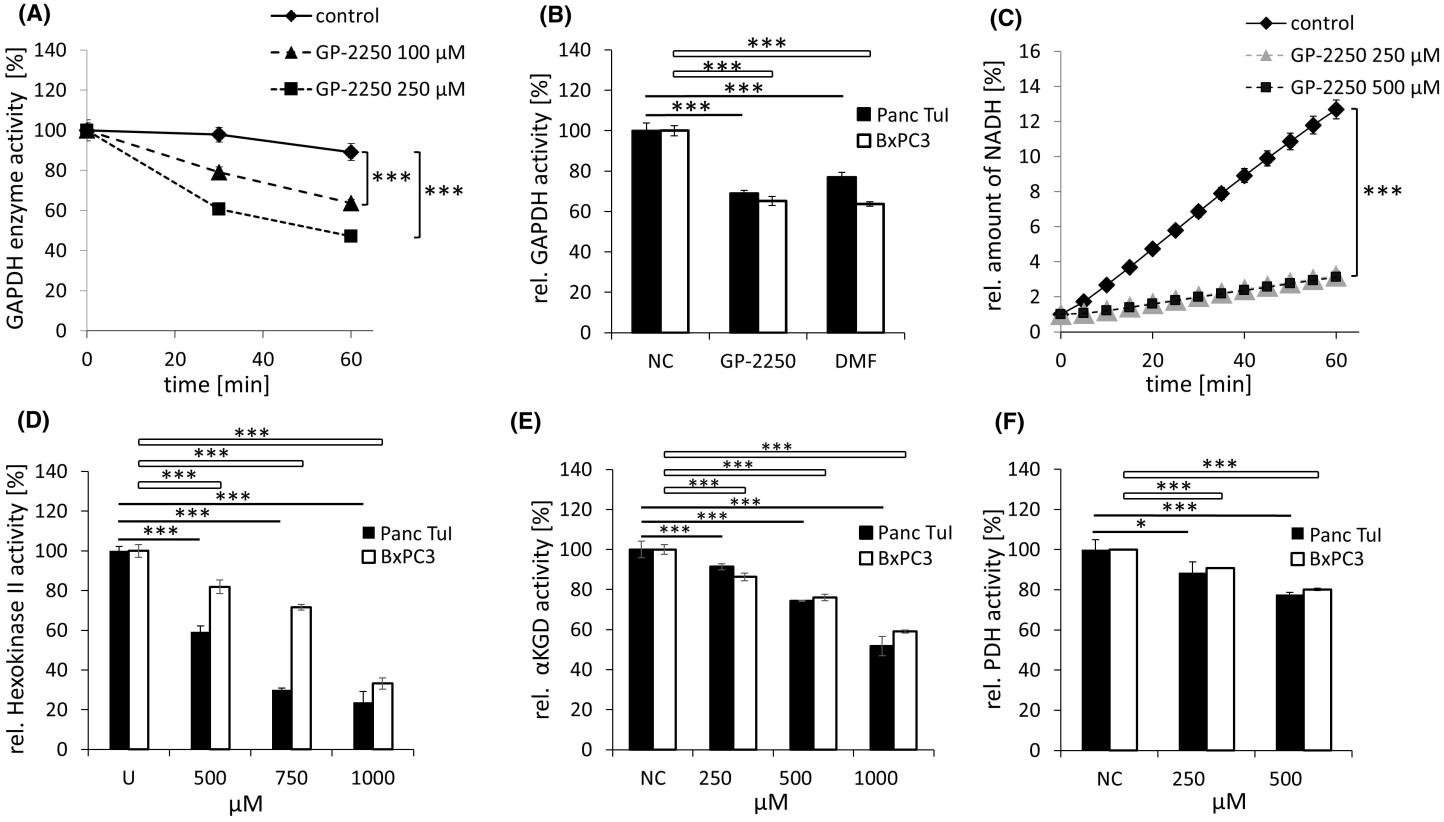
Inhibition of glycolytic and TCA cycle‐related enzymes. (A) Recombinant human GAPDH treated with 100 and 250 μM GP‐2250 for 30 and 60 min, followed by GAPDH enzyme activity assay. (B) Inhibition of GAPDH activity in Panc Tul and BxPC3 cells following incubation with GP‐2250 (500 μM) for 24 h. The GAPDH inhibitor DMF, 100 μM, was included as control. (C) Human recombinant hexokinase 2 (HK2) incubated with 250 and 500 μM GP‐2250 for 60 min. Formation of NADH was measured every 5 min. (D) Inhibition of hexokinase 2 (HK2)activity in Panc Tul and BxPC3 cells following incubation with GP‐2250 (250, 500 and 1000 μM) for 24 h. (E) Inhibition of alpha‐ketoglutarate dehydrogenase (αKGDH) following treatment of Panc Tul and BxPC3 cells for 24 h with GP‐2250 (250, 500 and 1000 μM). (F) Inhibition of pyruvate dehydrogenase (PDH) following treatment of Panc Tul and BxPC3 cells for 24 h with GP‐2250 (250 and 500 μM). Concentrations of GP‐2250 ranging from 100 to 1000 μM were used in the cellular assays of GAPDH, HK2, αKGDH and PDH, Shown are the results of the lowest effective concentrations, respectively. *p ≤ 0.05, significant; **p ≤ 0.01, highly significant; ***p ≤ 0.001, extremely significant. DMF, dimethylfumarate; GAPDH, glyceraldehyde‐3‐phosphate‐dehydrogenase; HK2, hexokinase 2; NADH, nicotinamide adenine dinucleotide + hydrogen; NC, negative control; U, untreated.

To assess the impact of GP‐2250 on glucose metabolism, HK2 and GAPDH activity was tested. HK2 was inhibited by 40.7% ± 2.9% and 18.1% ± 1.4% following the incubation of Panc Tul and BxPC3 cells, respectively, with GP‐2250 (500 μM) and further inhibited at higher concentrations (750, 1000 μM) (Figure 2D). Similarly, human recombinant HK2 was inhibited by 80.8% and 81.5% versus control following incubation for 60 min with 250 and 500 μM GP‐2250, respectively. (Figure 2C).

GAPDH was inhibited by 30.9% ± 1.3% and 34.7% ± 2.3% following the incubation of Panc Tul and BxPC3 cells, respectively, with GP‐2250 (500 μM) (Figure 2B). The selective GAPDH inhibitor dimethylfumarate (DMF) was included as reference.^18^ Likewise, recombinant human GAPDH (rhGAPDH) activity was reduced by 20% and 40%, respectively, versus control following incubation for 30 min with 100 and 250 μM GP‐2250 (Figure 2A). Inhibition of rhGAPDH was further increased at 60 min incubation, although the measurements were impacted by the known natural loss of activity of rhGAPDH. Besides HK2 and GAPDH, PDH and αKGDH were dose‐dependently inhibited by GP‐2250. PDH was reduced by 11.6% ± 0.6% at 250 μM and 22.4% ± 0.3% at 500 μM in Panc TuI; 9.3% ± 1.8% at 250 μM and 19.8% ± 4.6% at 500 μM in BxPC3 (Figure 2F). αKGDH was reduced by 8.6% ± 0.2% at 250 μM; 25.7% ± 0.0% at 500 μM and 48.2% ± 0.1% at 1000 μM in Panc TuI; 13.6% ± 1.8% at 250 μM; 24.1% ± 1.6% at 500 μM and 40.9% ± 0.75% at 1000 μM in BxPC3 (Figure 2E).

**FIGURE 3:**
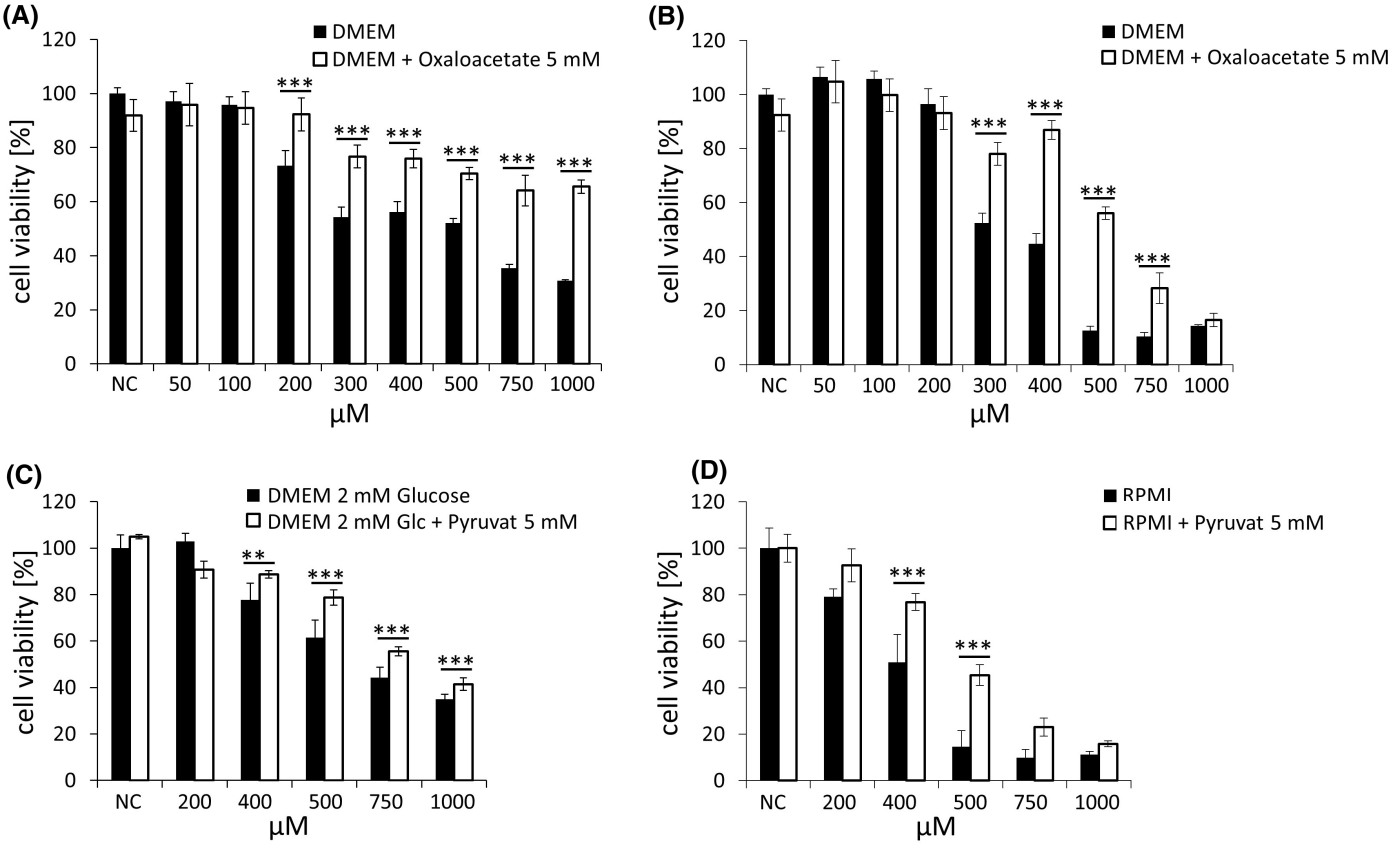
Rescue from GP‐2250‐induced cytotoxicity by oxaloacetate or pyruvate. (A) Panc Tul and (B) BxPC3 cells treated with different concentrations of GP‐2250 (50–1000 μM) for 24 h in the absence (black columns) or presence (white columns) of supplementary oxaloacetate (OAA, 5 mM). (C) Panc Tul and (D) BxPC3 cells were treated with different concentrations of GP‐2250 (100–1000 μM) for 24 h in the absence (black columns) or presence (white columns) of pyruvate (PYR 5 mM). Cell viability was tested with MTT. There was major significant protection from GP‐2250‐induced cytotoxicity by OAA and PYR nearly over the entire range of active drug concentrations. NC, negative control. *p ≤ 0.05, significant; **p ≤ 0.01, highly significant; ***p ≤ 0.001, extremely significant.

Rescue experiments with the TCA cycle components OAA and PYR supported the view that the energy deficit was causal for the cytotoxicity. Addition of OAA as a supplementary substrate strongly reduced the cytotoxicity over the entire concentration range of GP‐2250 (200–1000 μM) in both Panc Tul and BxPC3 cell lines (Figure 3A,B). Addition of PYR likewise reduced the cytotoxicity of GP‐2250 to a large extent over the entire active drug concentration range tested (300–1000 μM) (Figure 3C,D).

### 3.3 Generation of ROS

**FIGURE 4:**
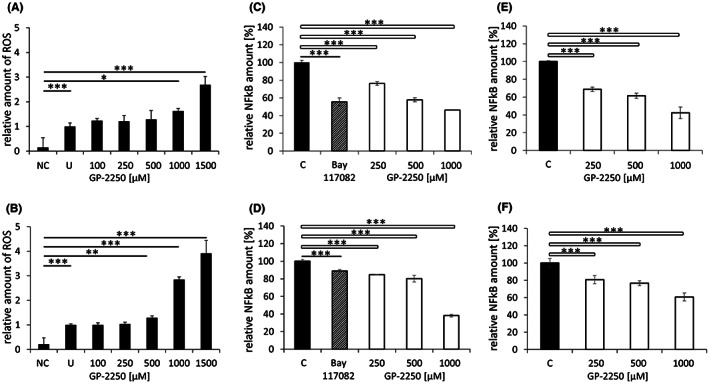
Generation of ROS and inhibition of NF‐κB. Generation of ROS in Panc TuI (A) and BxPC3 (B) cells following incubation with various concentrations of GP‐2250 for 90 min compared to an untreated control (UC). Negative control (NC) contained NAC (5 mM) in the presence of 1000 μM GP‐2250. Inhibition of NF‐kB (C, D) p65/DNA binding was dose‐dependently inhibited following incubation of Panc Tul and BxPC3 cells with GP‐2250 (250–1000 μM) for 24 h, as tested in lysates of the nuclear fraction. Bay 117082 (10 μM) served as control. (E, F) p65/DNA binding in lysates of the nuclear fraction from untreated Panc TuI and BxPC3 cells incubated directly with GP‐2250 (250, 500 and 1000 μM). C, non‐treated control; NAC, n‐acetylcysteine; NC, negative control; NF‐κB, nuclear factor kappa‐light‐chain‐enhancer of activated B cells; ROS, reactive oxygen species; SD, standard deviation; UC, untreated control. *p ≤ 0.05, significant; **p ≤ 0.01, highly significant; ***p ≤ 0.001, extremely significant.

In both Panc Tul and BxPC3 cells, a strong rise in ROS was already apparent after 90 min of incubation with higher concentrations of GP‐2250 (Figure 4A,B). Addition of the reducing agent NAC (n‐acetylcysteine) abrogated the rise in ROS as shown by the negative control (NC). The ROS level was slightly higher in the untreated control (UC), which was devoid of NAC, a finding which presumably reflects the ROS base level in the cells.

### 3.4 Activation of AMPK

**FIGURE 5:**
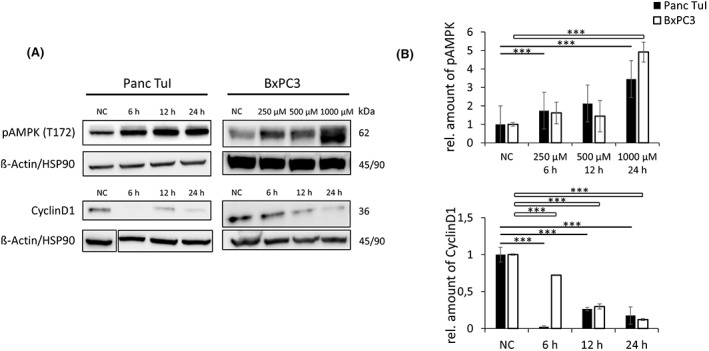
Representative western blot (A) and quantitative analysis (B) of pAMPK and cyclin D1. pAMPK: Time dependency of AMPK phosphorylation at T172 following incubation of Panc Tul with GP‐2250 (250 μM).) Dose‐dependency of AMPK phosphorylation after incubation of BxPC3 cells+ for 24 h. Cyclin D1: Time‐dependent decrease of cyclin D1 following incubation of Panc Tul (500 μM GP‐2250) and BxPC3 cells (250 μM GP‐2250). ß‐Actin/HSP‐90 used as internal controls. NC, negative control.

The energy deficit induced by GP‐2250 was expected to activate the metabolic regulator AMPK through phosphorylation at threonine 172 (Thr 172) by an upstream kinase.^11^, ^12^ In the presence of GP‐2250, the phosphorylation of AMPK at Thr 172 was increased in a time‐dependent manner (6–24 h) as tested in PancTul cells (Figure 5A). The dose‐dependency in the range of 250–1000 μM was determined in BxPC3 cells (Figure 5B). To test whether the activation of AMPK was functionally significant, two downstream targets were analysed, acetyl‐CoA carboxylase (ACC) and Raptor.

### 3.5 Phosphorylation of the rate‐limiting enzyme of fatty acid synthesis and Raptor

**FIGURE 6:**
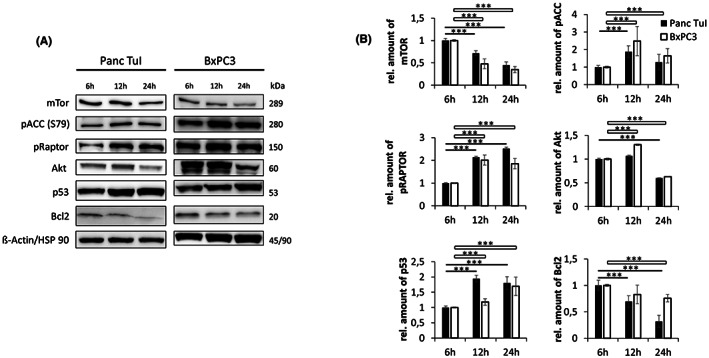
Representative western blot (A) and quantitative analysis (B) of regulatory proteins in Panc TuI (A) and BxPC3 (B) cells. pACC: Time‐dependent increase of ACC‐1 phosphorylation at Ser79 and of Raptor at Ser792 following incubation of Panc Tul (250 μM GP‐2250) and BxPC3 (500 μM GP‐2250). pRaptor: Time‐dependent Raptor phosphorylation at Ser 792 in PancTul and BxPc3 cells incubated with 500 and 1000 μM GP‐2250. p53: Time‐dependent increase of protein level of p53 in Panc Tul and BxPC3 cells (500 μM GP‐2250). Akt and mTor: Time‐dependent decrease of the protein level of Akt and mTor at 1000 μM GP‐2250 in Panc TuI and BxPC3 cells. Bcl2: Time‐dependent downregulation of protein level of Bcl2 following incubation of Panc Tul and BxPC3 cells (500 μM GP‐2250). ß‐Actin/HSP‐90 used as internal controls.

The enzyme ACC, the rate‐limiting step of fatty acid synthesis, is susceptible to AMPK‐induced inhibition through phosphorylation at serine 79 (Ser79). In the presence of GP‐2250, ACC‐1 was increasingly phosphorylated at Ser79 over time, which was apparent for both Panc TuI (250 μM) and BxPC3 (500 μM), as tested by western blotting (Figure 6). The phosphorylation was somewhat more prominent at 12 h than at 24 h. The phosphorylation of ACC‐1 points to a potential impact of GP‐2250 on fatty acid synthesis.

The mTOR complex 1 (mTORC1) drives cellular growth^23^, ^24^ and can be directly inhibited by AMPK through phosphorylation of its constituent protein Raptor at Ser792.^11^, ^24^, ^25^, ^26^

In the presence of GP‐2250 (500 μM), Raptor phosphorylation at Ser792 was time‐dependently increased as shown in western blots with Panc TuI cells responding at a lower drug concentration (500 μM) than BxPC3 (1000 μM) (Figure 6). In addition, the mTOR protein level was time‐dependently reduced in both cell lines by GP‐2250 (1000 μM) (Figure 6). The protein level of the upstream serine/threonine‐kinase Akt, a driver of tumour proliferation, was likewise time‐dependently decreased by GP‐2250 (1000 μM) in both cell lines (Figure 6). These results point to a possible inhibition of cell growth by GP‐2250.

### 3.6 Inhibition of the NF‐κB and its transcriptional activity

To test whether GP‐2250 might inhibit the NF‐kB pathway, p65/DNA binding was analysed in nuclear lysates prepared from Panc Tul and BxPC3 cells, which had been treated for 24 h with various concentrations of GP‐2250. GP‐2250 caused a concentration‐dependent inhibition of p65/DNA binding in both cell lines, which was significant already at 250 μM and reached 42.1% in Panc TuI (500 μM) and 19.7% in BxPC3 (500 μM). Bay 117082 served as control^18^ (Figure 4C,D). In addition, when lysates from untreated Panc Tul and BxPC3 cells were incubated with the same concentrations of GP‐2250, a comparable degree of inhibition of p65/DNA binding was found (Figure 4E,F). This result supports the view that GP‐2250 is able to directly inhibit p65/DNA binding.

The inhibition of p65/DNA binding by GP‐2250 resulted in a reduced expression of the downstream targets cyclin D1 and Bcl2. The expression of cyclin D1 was time‐dependently (6 h to 24 h) reduced by GP‐2250 as shown by western blotting for Panc Tul and BxPC3 cell lines (Figure 5A,B), being already apparent after 6 h of incubation. The expression of the anti‐apoptotic protein Bcl2 was likewise reduced in a time‐dependent manner in Panc Tul and BxPC3 cells as shown by western blotting (Figure 6). The time‐dependent reduction of cyclin D1 expression supports the mitosis‐inhibitory effect of GP‐2250^1^ and the Bcl2 deficit is expected to increase apoptosis.

### 3.7 Upregulation of p53

As a response to metabolic and oxidative stress, cells frequently respond by arresting cell division or by inducing apoptosis, triggered by the transcription factor and tumour repressor p53.^27^, ^28^ In the presence of GP‐2250 (500 μM), p53 protein levels were time‐dependently increased, serving as a sign of activation of the stress response (Figure 6).

## 4 DISCUSSION

### 4.1 Inhibition of energy metabolism causes anticancer activity

**FIGURE 7:**
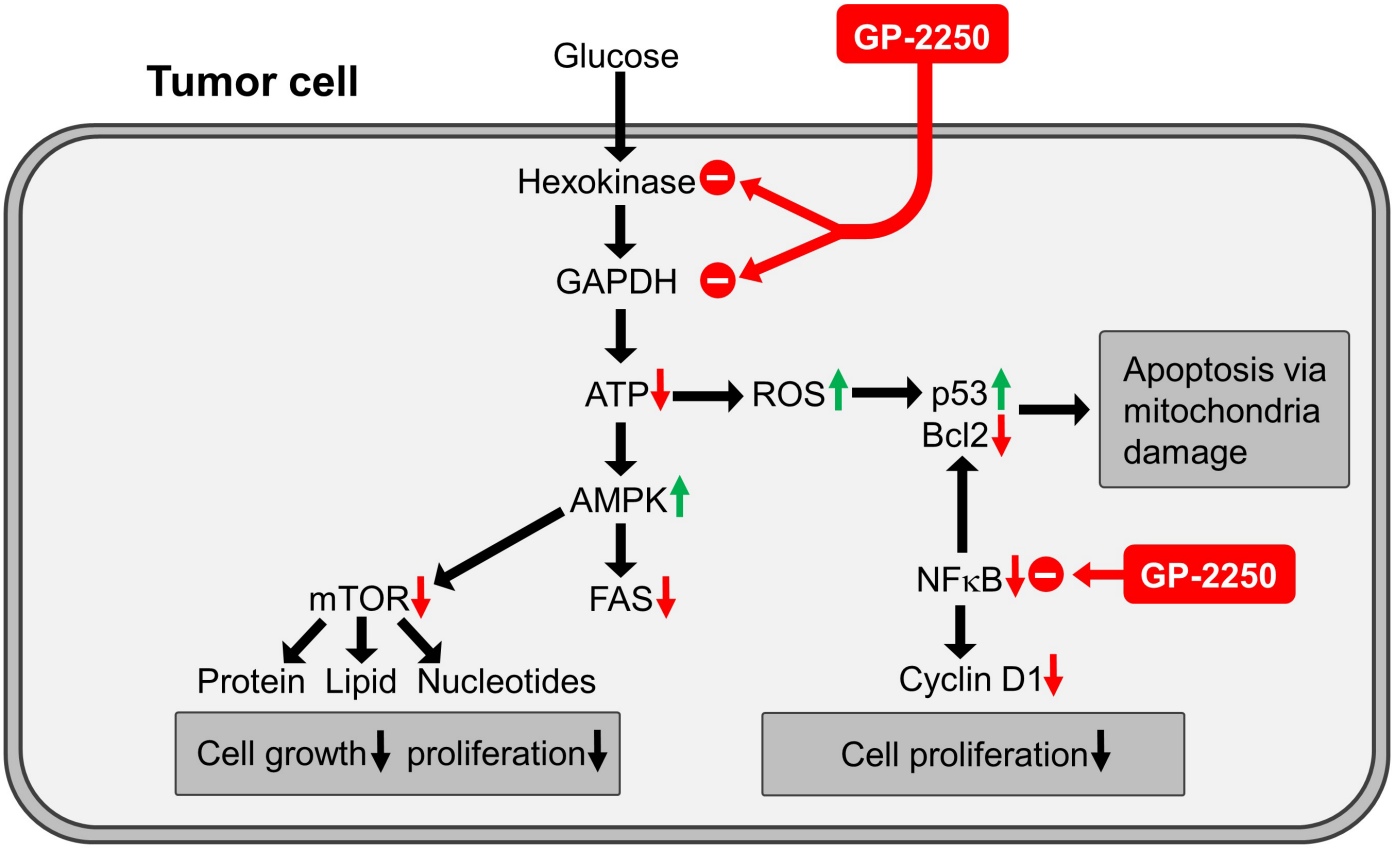
Proposed scheme of the alteration of cancer cell metabolism and NF‐κB inhibition by GP‐2250. Bold black arrows indicate metabolic pathways; red and green arrows indicate drug‐induced changes. By limiting the energy metabolism through the inhibition of hexokinase 2 and GAPDH, GP‐2250 induces an energy deficit in line with an impairment of the TCA cycle. The reduction of ATP triggers the activation of the energy‐deficit sensor AMPK. Its downstream events include the inhibition of mTOR, a major driver of tumour cell growth and potential impairment of fatty acid synthesis (FAS) through ACC‐1 inhibition. The inhibition of NF‐κB by GP‐2250 limits the rate of tumour cell proliferation through cyclin D1 downregulation. It also promotes apoptosis through downregulation of the anti‐apoptotic Bcl2. ROS contributes to the upregulation of the transcription factor p53, which supports apoptosis. AMPK, adenosine monophosphate‐dependent protein kinase; FAS, fatty acid synthesis; NF‐κB, nuclear factor kappa‐light‐chain‐enhancer of activated B cells; PDH, pyruvate dehydrogenase.

As outlined in a schematic overview (Figure 7), the anticancer agent GP‐2250 induced global alterations in tumour cell metabolism. Inhibition of glycolysis through inhibition of HK2 and GAPDH appears to be the prime event (Figure 2B,D), as these two enzymes are considered to be direct drug targets as demonstrated by the inhibition of the respective recombinant enzymes by GP‐2250 (Figure 2A,C). At comparable concentrations of GP‐2250 (500 μM), the TCA cycle enzymes αKGDH and PDH were inhibited to a lower degree than HK2 and GAPDH (Figure 2E,F), limiting their impact relative due to the inhibition of the upstream glycolysis.

The causative link between the cytotoxicity of GP‐2250 and the inhibition of the glycolytic enzymes and the TCA cycle was demonstrated in two rescue experiments. The ability of supplementary OAA and PYR to largely rescue tumour cells from drug‐induced cytotoxicity is evidence that the deficit in glycolytic and TCA cycle‐dependent energy metabolism is the major cause for the cytotoxicity of GP‐2250 (Figure 3A–D).

Both HK2 and GAPDH are upregulated in many human tumours and serve as drug targets.^8^ Inhibitors of HK2 such as benitrobenrazide show anticancer activity as demonstrated in tumour xenograft models^29^ and clinical trials have started with the HK2 inhibitor lonidamine.^8^ Apart from limiting glycolysis, HK2 inhibition is expected to contribute to the rise of ROS by reducing the synthesis of the reducing agent NADPH through inhibition of the pentose phosphate pathway.^5^, ^30^

The selective GAPDH inhibitors koningic acid^9^ and DMF^18^, ^31^ displayed cytotoxicity in a multitude of cancer cell lines in vitro and in vivo, including colon cancer cells,^32^ breast cancer cells,^33^ melanoma cells^34^ and hepatocellular carcinoma.^35^

PDH is the gatekeeper between glycolysis and the TCA cycle,^36^ and the inhibition of the TCA cycle itself by αKGDH inhibition is known to contribute to an energy deficit and support anticancer and anti‐metastatic activity as shown for the αKGDH inhibitors CPI‐613 and AA6, respectively.^37^, ^38^ These examples illustrate that the inhibition of aerobic glycolysis and the TCA cycle can induce anticancer activity. GP‐2250 impairs the energy metabolism by directly targeting the initial steps of glycolysis.

### 4.2 Activation of AMPK

The impairment of energy metabolism by GP‐2250 was most clearly apparent by the decrease of ATP (Figure 1B,C), which represents a threat to survival of the tumour cells. As a countermeasure to preserve ATP, the energy‐deficit sensor AMPK was activated as shown by the time‐dependent phosphorylation of the AMPK alpha subunit at threonine 172 in Panc Tul cells (Figure 5A) and the dose dependency of the phosphorylation of AMPK in BxPC3 cells (Figure 5B). AMPK activation is known to inhibit major downstream ATP‐consuming biosynthetic pathways required for cell growth and proliferation, including the synthesis of proteins, fatty acids, cholesterol, nucleotides, ribosomal RNA and glycogen.^11^, ^12^ Due to this austerity programme, AMPK activation suppresses tumour growth.^10^ The activation of AMPK is therefore a hallmark of the anticancer activity of GP‐2250.

### 4.3 Inhibition of ACC

AMPK inhibits lipogenic transcriptional programmes including the synthesis of fatty acids. This is in keeping with the phosphorylation of ACC‐1 in the presence of GP‐2250 (Figure 6), pointing to a potential deficit of fatty acid synthesis in Panc Tul and BxPC3 cells. The power of ACC inhibition in suppressing the growth of cancers is underlined by the finding that drug‐induced ACC phosphorylation or a specific ACC inhibitor was effective in suppressing hepatocellular carcinoma and pancreatic cancer.^39^, ^40^ Thus, the cytotoxicity of GP‐2250 may be attributed, at least in part, to a lack of fatty acid synthesis. The ability of supplementary OAA to rescue Panc Tul and BxPC3 cells from GP‐2250‐induced cytotoxicity (Figure 3A,B) might be attributed, at least in part, to a possible restoration of fatty acid synthesis. An excess of OAA is likely to override the ACC inhibition by increasing the transport capacity to provide mitochondrial Acetyl‐CoA as substrate for the cytosolic ACC.

### 4.4 Inhibition of RAPTOR/mTOR

AMPK regulates protein synthesis in large part by inhibition of the mTORC1 complex, a key driver of tumour growth.^24^, ^41^ The time‐dependent increase of RAPTOR phosphorylation at Ser792 by GP‐2250 (Figure 6), suggests that GP‐2250 is able to inhibit protein synthesis. There are reciprocal interactions between mTOR and Akt. The protein kinase Akt also drives tumour growth and proliferation. At high concentrations of GP‐2250, the protein levels of both mTOR and Akt were reduced, supporting the view that GP‐2250 inhibits protein synthesis (Figure 6). Besides inhibiting the activity of mTOR through RAPTOR phosphorylation, AMPK can also limit protein synthesis indirectly via TSC2 and directly by blocking RNA synthesis and protein elongation.^11^, ^12^

### 4.5 A lack of nucleotides?

As part of the downstream events of AMPK activation, the synthesis of nucleotides can be switched off by inhibition of PRPP (phosphoribosyl‐1‐pyrophosphate)‐synthase.^11^ Although not tested experimentally, an inhibition of nucleotide synthesis by GP‐2250 would be in keeping with the rescue experiment performed with OAA (Figure 3A,B). The synthesis of both pyrimidine‐ and purine‐nucleotides requires aspartate.^5^, ^7^ As a precursor to aspartate, supplementary OAA may be able to restore the synthesis of nucleotides by replenishing aspartate and override PRPP inhibition. As nucleotides are essential building blocks for the formation of RNA and DNA, it cannot be excluded that impaired DNA and RNA synthesis is part of the cytotoxic effects of GP‐2250.

A deficiency in nucleotide synthesis would be expected to also reduce the level of nicotinamide adenine dinucleotide (NAD+). Among other functions, NAD serves as a substrate for the enzyme PARP [poly (ADP‐ribose) polymerase], which is required for single strand homologous recombination repair. As PARP inhibitors are used for the treatment of ovarian and other cancers, it is of interest that GP‐2250 might act as an indirect, metabolic PARP inhibitor by limiting the availability of the substrate NAD+.

### 4.6 Inhibition of the NF‐κB pathway

NF‐κB ensures survival of the tumour cell in several ways. It enhances cell cycle progression by upregulating cyclin D1, prevents apoptosis by upregulating the anti‐apoptotic protein Bcl2 and protects cells from toxic ROS levels by upregulating antioxidant enzymes such as SOD1 and HMOX1.^42^ The cancer drugs tacrolimus and bortezomib act, at least in part, by suppressing NF‐κB.^43^, ^44^ GP‐2250 was found to act as NF‐kB inhibitor based on its inhibition of p65/DNA binding in nuclear lysates. This was apparent not only in nuclear lysates prepared from cells treated with GP‐2250 (Figure 4C,D), but also in nuclear lysates prepared from untreated cells with subsequent incubation of the lysate with GP‐2250 (Figure 4E,F). It is of note, that the dose dependency for p65/DNA binding in treated lysates was comparable to that from the lysates of treated cells. This finding suggests that GP‐2250 is likely to directly interfere with the binding of p65 to DNA and not with an upstream event.

A reduction of the transcriptional activity of NF‐κB was apparent in both Panc Tul and BxPC3 cell lines by the change in expression of cyclin D1 and Bcl2. This is testimony to the functional relevance of NF‐kB inhibition by GP‐2250. The expression of cyclin D1, the driver of cell cycle progression, was reduced by GP‐2250 (Figure 5). This finding is in line with the previously shown reduction of the rate of cell proliferation by GP‐2250.^1^ The expression of the anti‐apoptotic protein Bcl2 was likewise reduced by GP‐2250 (Figure 6). Thus, by inhibiting NF‐κB, GP‐2250 is able to disrupt tumour progression in a two‐pronged manner, by reducing the rate of cell proliferation and promoting apoptosis.

Several limitations of this study must be taken into account. The present study is exclusively an in vitro analysis using pancreatic cancer cell line models. The impact of GP‐2250 on growth control via lipid and protein synthesis requires further study. Whether the present findings can be confirmed in an analysis of pancreatic cancer tissue in mouse PDX models remains to be seen. Further studies are also required to test whether the mechanism of action described here extends to other types of tumours.

## 5 CONCLUSION

The anticancer agent GP‐2250, presently in clinical development, acts by disrupting the energy metabolism through direct inhibition of HK2, GAPDH and an impact on the TCA cycle. This view is supported by the ability of supplementary OAA and PYR to protect the tumour cells to a large extent from drug‐induced cytotoxicity. Activation of the energy‐deficit sensor AMPK is attributed to the deficit of ATP. Its downstream events include the inhibition of ACC, the rate‐limiting enzyme of fatty acid synthesis and inhibition of the Raptor/mTor complex, both of which limit the synthesis of essential cell components. In addition to metabolic deficits, the transcriptional activity of the tumour promoter NF‐κB is inhibited by GP‐2250. The downregulation of cyclin D1 and the anti‐apoptotic Bcl2 is in line with a reduction of the rate of tumour cell proliferation and induction of apoptosis, respectively. The upregulation of p53 concomitant with the rise of ROS further contributes to cytotoxicity. These metabolic and transcriptional findings provide a molecular basis for the ability of GP‐2250 to reduce tumour cell proliferation and induce apoptotic cell death as shown here for pancreatic cancer cells. The effectiveness of GP‐2250 in patient‐derived xenograft mouse models and its role in combination with approved anticancer drugs, such as gemcitabine, is under investigation.

## AUTHOR CONTRIBUTIONS

**Britta Majchrzak‐Stiller:** Conceptualization (equal); data curation (equal); formal analysis (equal); methodology (equal); visualization (equal); writing – original draft (equal). **Marie Buchholz:** Conceptualization (equal); data curation (equal); formal analysis (equal); methodology (equal); visualization (equal); writing – original draft (equal). **Ilka Peters:** Investigation (equal); validation (equal); writing – review and editing (equal). **Daniel Waschestjuk:** Investigation (equal); validation (equal). **Johanna Strotmann:** Investigation (equal); validation (equal). **Philipp Höhn:** Conceptualization (equal); funding acquisition (equal); writing – original draft (equal). **Stephan Hahn:** Investigation (equal); resources (equal); writing – review and editing (equal). **Chris Braumann:** Conceptualization (equal); funding acquisition (equal); supervision (equal); writing – review and editing (equal). **Waldemar Uhl:** Conceptualization (equal); funding acquisition (equal); resources (equal). **Thomas Müller:** Resources (equal); writing – review and editing (equal). **Hanns Möhler:** Conceptualization (equal); project administration (equal); supervision (equal); writing – original draft (equal); writing – review and editing (equal).

## CONFLICT OF INTEREST STATEMENT

BM‐S, MB, IP, DW, JS, PH, WU are employees of Department of General and Visceral Surgery, St. Josef‐Hospital, Germany, which received research funding from Geistlich Pharma AG to conduct the study.HM received consultancy fees from Geistlich Pharma AG. TM is employed by Geistlich Pharma AG, Wolhusen, Switzerland (Geistlich). Beyond the contribution of one author (who provided chemical expertise of the analysed agent) Geistlich had no role in the design of the study; in the collection, analyses, or interpretation of data; in the writing of the manuscript; or in the decision to publish the results. All other authors declare no competing interests.

## Data Availability

The data presented in this study are available on request from the corresponding author.

## References

[1] Buchholz M , Majchrzak‐Stiller B , Hahn S , et al. Innovative substance 2250 as a highly promising anti‐neoplastic agent in malignant pancreatic carcinoma – in vitro and in vivo. BMC Cancer. 2017;17:216. 28340556 10.1186/s12885-017-3204-x PMC5366103

[2] Buchholz M , Strotmann J , Majchrzak‐Stiller B , et al. New therapy options for neuroendocrine carcinoma of the pancreas‐the emergent substance GP‐2250 and gemcitabine prove to Be highly effective without the development of secondary resistances in vitro and in vivo. Cancer. 2022;14:2685. 10.3390/cancers14112685 PMC9179328 35681665

[3] Geistlich Pharma AG , Translational Drug Development . A phase 1/2 trial of GP‐2250 in combination with gemcitabine in pancreatic adenocarcinoma after FOLFIRINOX Chemotherapy: NCT03854110, GP‐2250‐1001. Available at: https://clinicaltrials.gov/ct2/show/NCT03854110; 2021 [Accessed 12.11.2021].

[4] Baron C , Buchholz M , Majchrzak‐Stiller B , et al. Substance GP‐2250 as a new therapeutic agent for malignant peritoneal mesothelioma‐a 3‐D In vitro study. Int J Mol Sci. 2022;23:7293. 35806313 10.3390/ijms23137293 PMC9267014

[5] DeBerardinis RJ , Chandel NS . Fundamentals of cancer metabolism. Sci Adv. 2016;2:e1600200. 27386546 10.1126/sciadv.1600200 PMC4928883

[6] Locasale JW , Cantley LC . Metabolic flux and the regulation of mammalian cell growth. Cell Metab. 2011;14:443‐451. 21982705 10.1016/j.cmet.2011.07.014 PMC3196640

[7] Vander Heiden MG , Cantley LC , Thompson CB . Understanding the Warburg effect: the metabolic requirements of cell proliferation. Science (New York, N.Y.). 2009;324:1029‐1033. 19460998 10.1126/science.1160809 PMC2849637

[8] Ciscato F , Ferrone L , Masgras I , Laquatra C , Rasola A . Hexokinase 2 in cancer: a prima Donna playing multiple characters. Int J Mol Sci. 2021;22:4716. 33946854 10.3390/ijms22094716 PMC8125560

[9] Liberti MV , Dai Z , Wardell SE , et al. A predictive model for selective targeting of the Warburg effect through GAPDH inhibition with a natural product. Cell Metab. 2017;26:648‐659.e8. 28918937 10.1016/j.cmet.2017.08.017 PMC5629112

[10] Faubert B , Boily G , Izreig S , et al. AMPK is a negative regulator of the Warburg effect and suppresses tumor growth in vivo. Cell Metab. 2013;17:113‐124. 23274086 10.1016/j.cmet.2012.12.001 PMC3545102

[11] González A , Hall MN , Lin S‐C , Hardie DG . AMPK and TOR: the yin and Yang of cellular nutrient sensing and growth control. Cell Metab. 2020;31:472‐492. 32130880 10.1016/j.cmet.2020.01.015

[12] Garcia D , Shaw RJ . AMPK: mechanisms of cellular energy sensing and restoration of metabolic balance. Mol Cell. 2017;66:789‐800. 28622524 10.1016/j.molcel.2017.05.032 PMC5553560

[13] Salminen A , Hyttinen JMT , Kaarniranta K . AMP‐activated protein kinase inhibits NF‐κB signaling and inflammation: impact on healthspan and lifespan. J Mol Med (Berl). 2011;89:667‐676. 21431325 10.1007/s00109-011-0748-0 PMC3111671

[14] Tan MH , Nowak NJ , Loor R , et al. Characterization of a new primary human pancreatic tumor line. Cancer Invest. 1986;4:15‐23. 3754176 10.3109/07357908609039823

[15] Kalthoff H , Schmiegel W , Roeder C , et al. p53 and K‐RAS alterations in pancreatic epithelial cell lesions. Oncogene. 1993;8:289‐298. 8426738

[16] Moritz CP . 40 years Western blotting: a scientific birthday toast. J Proteomics. 2020;212:103575. 31706026 10.1016/j.jprot.2019.103575

[17] Anderson M , Marayati R , Moffitt R , Yeh JJ . Hexokinase 2 promotes tumor growth and metastasis by regulating lactate production in pancreatic cancer. Oncotarget. 2017;8:56081‐56094. 28915575 10.18632/oncotarget.9760 PMC5593546

[18] Kornberg MD , Bhargava P , Kim PM , et al. Dimethyl fumarate targets GAPDH and aerobic glycolysis to modulate immunity. Science (New York, N.Y.). 2018;360:449‐453. 29599194 10.1126/science.aan4665 PMC5924419

[19] Liu X , Liu X , Bai J , et al. Glyceraldehyde‐3‐phosphate dehydrogenase restricted in cytoplasmic location by viral GP5 facilitates porcine reproductive and respiratory syndrome virus replication via its glycolytic activity. J Virol. 2021;95:e0021021. 34160254 10.1128/JVI.00210-21 PMC8387053

[20] Tretter L , Adam‐Vizi V . Alpha‐ketoglutarate dehydrogenase: a target and generator of oxidative stress. Philos Trans R Soc Lond B Biol Sci. 2005;360:2335‐2345. 16321804 10.1098/rstb.2005.1764 PMC1569585

[21] Joaquín‐Ovalle FM , Guihurt G , Barcelo‐Bovea V , et al. Oxidative stress‐ and autophagy‐inducing effects of PSI‐LHCI from Botryococcus braunii in breast cancer cells. Biotech (Basel). 2022;11:9. 35822782 10.3390/biotech11020009 PMC9264392

[22] Mantsounga CS , Lee C , Neverson J , et al. Macrophage IL‐1β promotes arteriogenesis by autocrine STAT3‐ and NF‐κB‐mediated transcription of pro‐angiogenic VEGF‐A. Cell Rep. 2022;38:110309. 35108537 10.1016/j.celrep.2022.110309 PMC8865931

[23] Saxton RA , Sabatini DM . mTOR signaling in growth, metabolism, and disease. Cell. 2017;168:960‐976. 28283069 10.1016/j.cell.2017.02.004 PMC5394987

[24] Pópulo H , Lopes JM , Soares P . The mTOR signalling pathway in human cancer. Int J Mol Sci. 2012;13:1886‐1918. 22408430 10.3390/ijms13021886 PMC3291999

[25] Mihaylova MM , Shaw RJ . The AMPK signalling pathway coordinates cell growth, autophagy and metabolism. Nat Cell Biol. 2011;13:1016‐1023. 21892142 10.1038/ncb2329 PMC3249400

[26] Gwinn DM , Shackelford DB , Egan DF , et al. AMPK phosphorylation of raptor mediates a metabolic checkpoint. Mol Cell. 2008;30:214‐226. 18439900 10.1016/j.molcel.2008.03.003 PMC2674027

[27] Thoreen CC , Sabatini DM . AMPK and p53 help cells through lean times. Cell Metab. 2005;1:287‐288. 16054073 10.1016/j.cmet.2005.04.009

[28] Yogosawa S , Yoshida K . Tumor suppressive role for kinases phosphorylating p53 in DNA damage‐induced apoptosis. Cancer Sci. 2018;109:3376‐3382. 30191640 10.1111/cas.13792 PMC6215896

[29] Stine ZE , Schug ZT , Salvino JM , Dang CV . Targeting cancer metabolism in the era of precision oncology. Nat Rev Drug Discov. 2022;21:141‐162. 34862480 10.1038/s41573-021-00339-6 PMC8641543

[30] Pavlova NN , Thompson CB . The emerging hallmarks of cancer metabolism. Cell Metab. 2016;23:27‐47. 26771115 10.1016/j.cmet.2015.12.006 PMC4715268

[31] Saidu B , Edward N , Bretagne M , et al. Dimethyl fumarate is highly cytotoxic in KRAS mutated cancer cells but spares non‐tumorigenic cells. Oncotarget. 2018;9:9088‐9099. 29507676 10.18632/oncotarget.24144 PMC5823659

[32] Kaluzki I , Hailemariam‐Jahn T , Doll M , et al. Dimethylfumarate inhibits colorectal carcinoma cell proliferation: evidence for cell cycle arrest, apoptosis and autophagy. Cells. 2019;8:1329. 31661890 10.3390/cells8111329 PMC6912700

[33] Kastrati I , Siklos MI , Calderon‐Gierszal EL , et al. Dimethyl fumarate inhibits the nuclear factor κB pathway in breast cancer cells by covalent modification of p65 protein. J Biol Chem. 2016;291:3639‐3647. 26683377 10.1074/jbc.M115.679704 PMC4751401

[34] Takeda T , Tsubaki M , Asano R , et al. Dimethyl fumarate suppresses metastasis and growth of melanoma cells by inhibiting the nuclear translocation of NF‐κB. J Dermatol Sci. 2020;99:168‐176. 32693971 10.1016/j.jdermsci.2020.07.004

[35] Ganapathy‐Kanniappan S , Kunjithapatham R , Geschwind J‐F . Glyceraldehyde‐3‐phosphate dehydrogenase: a promising target for molecular therapy in hepatocellular carcinoma. Oncotarget. 2012;3:940‐953. 22964488 10.18632/oncotarget.623 PMC3660062

[36] Woolbright BL , Rajendran G , Harris RA , Taylor JA . Metabolic flexibility in cancer: targeting the pyruvate dehydrogenase kinase:pyruvate dehydrogenase Axis. Mol Cancer Ther. 2019;18:1673‐1681. 31511353 10.1158/1535-7163.MCT-19-0079

[37] Stuart SD , Schauble A , Gupta S , et al. A strategically designed small molecule attacks alpha‐ketoglutarate dehydrogenase in tumor cells through a redox process. Cancer & Metabolism. 2014;2:4. 24612826 10.1186/2049-3002-2-4 PMC4108059

[38] Atlante S , Visintin A , Marini E , et al. α‐Ketoglutarate dehydrogenase inhibition counteracts breast cancer‐associated lung metastasis. Cell Death Dis. 2018;9:756. 29988033 10.1038/s41419-018-0802-8 PMC6037705

[39] Lally JSV , Ghoshal S , DePeralta DK , et al. Inhibition of acetyl‐CoA carboxylase by phosphorylation or the inhibitor ND‐654 suppresses lipogenesis and hepatocellular carcinoma. Cell Metab. 2019;29:174‐182.e5. 30244972 10.1016/j.cmet.2018.08.020 PMC6643297

[40] Gao L , Xu Z , Huang Z , et al. CPI‐613 rewires lipid metabolism to enhance pancreatic cancer apoptosis via the AMPK‐ACC signaling. Exp Clin Cancer Res. 2020;39:73. 10.1186/s13046-020-01579-x PMC7187515 32345326

[41] Dancey J . mTOR signaling and drug development in cancer. Nat Rev Clin Oncol. 2010;7:209‐219. 20234352 10.1038/nrclinonc.2010.21

[42] Karin M . Nuclear factor‐kappaB in cancer development and progression. Nature. 2006;441:431‐436. 16724054 10.1038/nature04870

[43] Vafadari R , Kraaijeveld R , Weimar W , Baan CC . Tacrolimus inhibits NF‐κB activation in peripheral human T cells. PLoS One. 2013;8:e60784. 23573283 10.1371/journal.pone.0060784 PMC3613409

[44] Zavrski I , Jakob C , Kaiser M , Fleissner C , Heider U , Sezer O . Molecular and clinical aspects of proteasome inhibition in the treatment of cancer. Recent Results Cancer Res. 2007;176:165‐176. 17607924 10.1007/978-3-540-46091-6_14

